# Resequencing and Transcriptome Analyses Reveal Variations and Expression Patterns of the RR Gene Family in Cucumber

**DOI:** 10.3390/genes16040409

**Published:** 2025-03-31

**Authors:** Ke Su, Wenhong Ao, Zhaolong Sun, Jing Li, Yu Gao, Defang Gan, Jingjing Yang

**Affiliations:** 1 School of Horticulture, Anhui Agricultural University, Hefei 230036, China; 15856829404@163.com (K.S.); Long5656566@126.com (Z.S.); Ljing6297@stu.ahau.edu.cn (J.L.); 2 Beijing Academy of Agriculture and Forestry Science, Beijing 100097, China; whao202304@126.com (W.A.); zfanqie6@gmail.com (Y.G.)

**Keywords:** cucumber, CsRR, variation, transcriptome, *Fusarium oxysporum* f. sp. *Cucumer-inum* (Foc)

## Abstract

Background: Cucumber (*Cucumis sativus* L.) is an important economic crop worldwide. Response regulators (RRs) play crucial roles in plant growth, development, and responses to both biotic and abiotic stresses. Methods: Combined analysis of 182 re-sequencing and transcriptome datasets was conducted to investigate *CsRR* variations, with subsequent RT-qPCR experiments confirming its functional significance. Results: In this study, 18 *CsRR* genes were identified and classified into three groups according to their protein structures: A-ARRs (3), B-ARRs (8), and PRRs (7). Resequencing uncovered critical mutations (non-synonymous SNPs, frameshift, and stop-gain variants) in *CsRR* genes. Transcriptome data revealed that five genes responded to abiotic stress and four responded to biotic stress. *CsPRR1* was upregulated in both resistant and susceptible lines at five dpi, downregulated in resistant plants at nine dpi, and showed no significant difference at 11 dpi. *CsPRR2* was consistently upregulated in both lines at 5, 9, and 11 dpi. *CsPRR3* was upregulated in resistant lines at nine dpi but downregulated at 11 dpi. *CsARR8* was significantly downregulated in both lines at 9 and 11 dpi. Notably, *CsPRR2* demonstrated dual functionality related to (i) the regulation of immature fruit skin color via a stop-gain InDel and (ii) resistance to Foc, as the gene was upregulated in both resistant and susceptible lines after inoculation with the pathogen. Conclusions: This study integrated resequencing and transcriptomic data to comprehensively characterize *CsRR* genes, establishing a foundation for further exploration of their functional mechanisms in cucumber.

## 1. Introduction

Cucumber (*Cucumis sativus* L.) is an important economic crop worldwide [1]. According to the FAO, China produced 77.258 million tons of cucumbers in 2022, making it the top-ranking producer of these fruits globally and highlighting this crop’s significant agricultural, biological, and economic importance [2,3]. Based on geographic distribution, cucumber germplasm resources can be classified into four groups: Indian, East Asian, Eurasian, and Xishuangbanna (XSBN) [4].

Response regulators (RRs) are key proteins in plant development and stress responses [5]. They play crucial roles in phytohormone signaling, plant growth, and stress resistance [6] and can be classified into three subfamilies: (i) A-type authentic response regulators (ARRs), (ii) B-type ARRs, and (iii) pseudo-RRs (PRRs). A-type ARRs contain a receiver domain in the N-terminal region and a short variable extension in the C-terminal region [7,8]. They perform unique functions in plant growth and development, possibly regulated by cytokinin and proteasomes [9]. B-type RRs contain a receiver domain and an output domain with a large C-terminal extension [7,8,10]. In *Arabidopsis thaliana*, B-type ARR genes (*B-ARRs*) positively regulate cytokinin signaling [9]. PRRs contain N-terminal atypical pseudo-receptor domains and C-terminal CCT (CONSTANS, CO-like, and TOC1) domains [11]. *PRR* family genes play crucial roles in regulating the circadian clock, photoperiodic responses, and flowering time in *Arabidopsis*, serving as essential regulatory factors for plant growth, development, and environmental adaptation [12,13,14,15]. Recent studies have shown that the *PRR2* gene regulates chloroplast development and, in turn, alters fruit skin color in the Cucurbitaceae family [16,17].

Single nucleotide polymorphisms (SNPs) and insertions/deletions (InDels) are important genetic variants that are abundant in plant genomes and widely used in gene mapping, variety identification, and marker-assisted breeding [18,19,20]. These variants significantly impact plant phenotypes, including yield, disease resistance, and stress tolerance [21,22,23]. High-throughput marker detection technologies based on the use of SNPs have been developed, offering highly efficient marker detection at a low cost. Therefore, SNPs are referred to as third-generation molecular markers [24,25,26,27,28,29,30,31,32]. In contrast, InDels are well-suited for agarose gel electrophoresis and are cost-effective. They offer notable advantages in gene mapping and variety identification [33]. Specific types of mutations in SNPs, such as nonsynonymous changes, stop-gain, and stop-loss mutations, have significant effects on plant phenotypes, including resistance and yield [34,35]. Similarly, InDel mutations, including frameshift insertions, frameshift deletions, stop-gain, and stop-loss mutations, play critical roles in plant traits [36]. Several studies on cucumber have produced valuable resequencing data. For example, Zhang et al. reported the resequencing of 115 cucumber germplasm sources [37]; Yang et al. developed a database containing 182 cucumber germplasm sequences [38]; and Xu et al. published the resequencing of 219 cucumber germplasm lines [39]. These datasets revealed abundant genetic variants, such as SNPs and InDels, that are useful for cucumber research. However, no further analyses of the identified variants, particularly those in the *RR* gene family, have been reported using these datasets.

Although some *RR* genes have been elaborated in plants, there is no report of *CsRR* genes in resistance to abiotic stresses and biotic in cucumbers. Therefore, we systematically identified *RR* genes in the cucumber genome and analyzed the genetic variants using re-sequencing data from 182 cucumber accessions. By integrating transcriptome data from the Cucurbitaceae database, *CsRR* genes were found to be extensively involved in various growth and developmental processes and to play crucial roles in biotic and abiotic stress responses [40,41,42,43,44]. Further analyses identified four *CsRR* genes related to *Fusarium oxysporum* f. sp. *Cucumerinum* (Foc) resistance. Overall, *CsRR* genes were comprehensively characterized by integrating re-sequencing and transcriptome data, establishing a foundation for further exploration of their functional mechanisms.

## 2. Materials and Methods

### 2.1. Identification and Phylogenetic Analysis of CsRR Genes in Cucumber

To identify the *CsRR* family genes, the protein sequences of *RR* genes from *Arabidopsis* were downloaded from the TAIR database (https://www.arabidopsis.org/, accessed on 23 January 2025). The protein sequences of *AtRR* genes were aligned to the cucumber protein sequence using the BLASTp function in TBtools v2.154 [45]. Additionally, the Pfam [46] online database (http://pfam-legacy.xfam.org/, accessed on 25 January 2025) was used to obtain the hidden Markov model (HMM) file of the response regulator receiver domain (PF00072). Subsequently, Pfam [46] and the Conserved Domain Database (CDD) [47] (https://www.ncbi.nlm.nih.gov/guide/domains-structures/, accessed on 26 January 2025) were used to search the RR receiver domain. Finally, 18 *CsRR* genes were identified in the cucumber genome. MEGA 7.0 software was employed, using the neighbor-joining method with 1000 bootstrap replicates to construct a phylogenetic tree. Chromosome density information was extracted from the genome annotation files using TBtools v2.119. The protein sequences of the CsRR genes family are presented in Table S1.

### 2.2. Variant Identification and Annotation

The total variant database used in this study was the same as that available in the VegSNPDB (VegSNPDB, http://www.vegsnpdb.cn/, accessed on 26 February 2025) [38]. Variants located in *CsRR* genes were extracted from the database using an in-house Perl script. SNPs and InDels were annotated using the gene-based annotation module in ANNOVAR (version 20200316) [48]. Table S2 shows the number of genome-wide mutations and different types of mutations in the CsRR gene family.

### 2.3. Genetic Diversity Analysis

PIC (Polymorphism Information Content), MAF (Minor Allele Frequency), GD (genetic diversity), and Ho (observed heterozygosity) values were calculated using an in-house Perl script.

### 2.4. RNA-Seq Data Analysis

To analyze the expression of *CsRR* genes in various tissues and organs, transcriptome data for these genes were retrieved from the CuGenDBv2 database under the biological project PRJNA80169 (http://cucurbitgenomics.org/organism/2, accessed on 27 February 2025). In addition, previously published transcriptome sequencing data of cucumber under NaCl stress (PRJNA285071), chilling stress (PRJNA438923), high-temperature stress (PRJNA634519), and Foc stress (PRJNA472169) were obtained from the Cucurbit Genomics Database to explore the transcriptional profiles. To further investigate the functions of *CsRR* genes, all RNA-seq data in CuGenDBv2 with an FPKM (Fragments Per Kilobase of transcript per Million mapped reads) value > 20 were screened, with a ratio before and after treatment of >2. FPKM heat maps of *CsRR* genes were generated using TBtools v 2.119 software. The FPKM values are shown in Table S3.

### 2.5. Plant Materials, qRT-PCR Analysis of CsRR Genes After Foc Inoculation

The transcriptome analysis indicated that four *CsRR* genes were involved in resistance to Foc. To validate these results, the relative expression of these four genes was measured in cucumber germplasm after inoculation with Foc. The cucumber varieties WI2757 and JY-2 were inoculated with Foc using the bacterial soil method at a 10% mass ratio. The cucumber plants were cultivated in a light-controlled incubator under a 28 °C/16 °C 16 h/8 h day/night cycle. The cucumber roots were cleaned, and samples were taken from the root mid-section at 5, 9, and 11 days post-inoculation (dpi). The samples were frozen immediately in liquid nitrogen, with three replicates taken at each time point.

Total RNA was extracted using a FastPure Universal Plant Total RNA Isolation Kit (Vazyme, Beijing, China), and cDNA was synthesized from RNA using a PrimeScript^TM^ RT reagent Kit with gDNA Eraser (TaKaRa, Beijing, China). Quantitative real-time PCR (RT-qPCR) was performed on a Bio-Rad (CFX Opus 96, Beijing, China) Real-Time PCR system using TB Green Fast qPCR mix (TaKaRa, Beijing, China). The primers are listed in Supplementary Table S1, and the cycling conditions were as follows: 30 s at 95 °C, followed by 39 cycles of 5 s at 95 °C and 30 s at 60 °C, with a final step of 15 s at 95 °C. For each analysis, three technical replicates and three biological replicates were performed. Gene expression levels were calculated using the 2^−ΔΔCT^ method. The qRT-PCR primer sequences are listed in Table S4.

## 3. Results

### 3.1. The Identification of CsRR Genes

**Figure 1:**
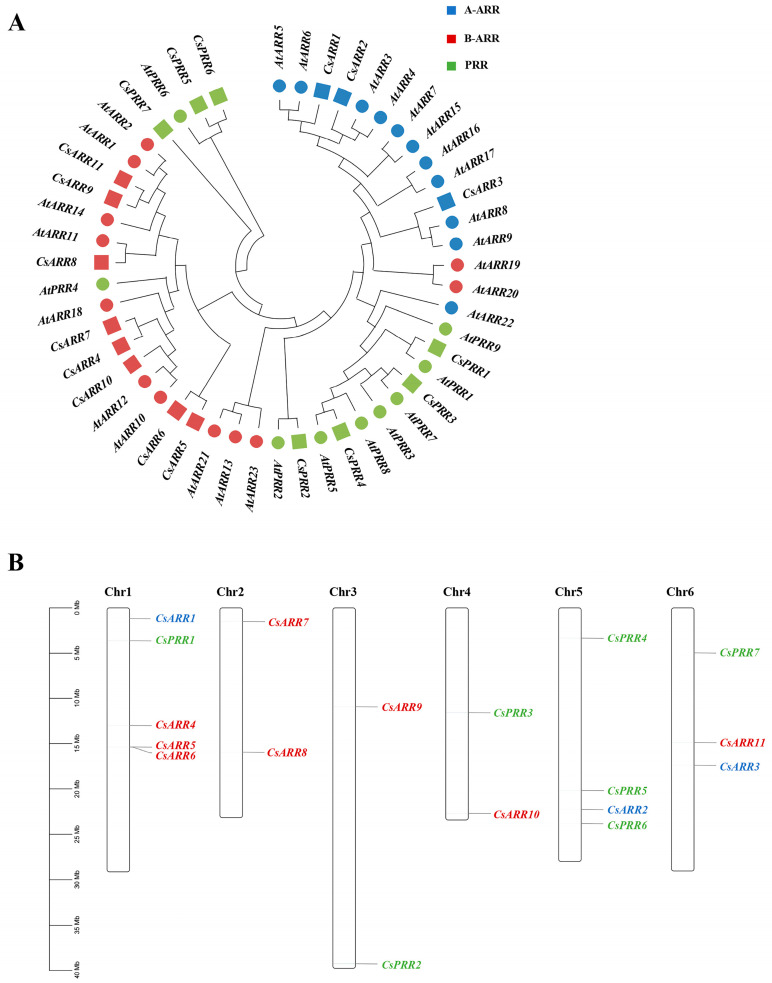
(**A**) The phylogenetic relationships between *Arabidopsis* (At) and *C. sativus* (Cs) Response regulator (*RR*) gene family members. (**B**) The chromosomal localization of 18 CsRR family genes. Blue, red, and green represent A-type authentic response regulator (*A-ARR*), B-type authentic response regulator (*B-ARR*), and pseudo-RR (*PRR*) genes, respectively.

*RR* gene family members were identified in the cucumber genome using BLASTp and hidden Markov model (HMM) searches. A total of 18 *CsRR* genes were identified and categorized into three groups: A-type (three members, *CsRR1*–*3*), B-type (eight members, *CsRR4*–*11*), and PRRs (seven members, *CsPRR1–7*). Creating a phylogenetic tree based on CsRR proteins revealed high homology between *CsRRs* and *AtRRs* (Figure 1A). The homology between *CsARR2* and *AtARR4* was the highest at 78.2%, while the lowest was between *CsPRR7* and *AtPRR9* at 9.7%. The *CsRR* genes were randomly distributed across six chromosomes, with none found on chromosome 7 (Figure 1B). Notably, chromosome 1 contained the most *CsRR* genes (five), while chromosomes 2, 3, and 4 contained the least, harboring two each.

### 3.2. SNP and InDel Variations in 182 Cucumber Germplasms

**Figure 2:**
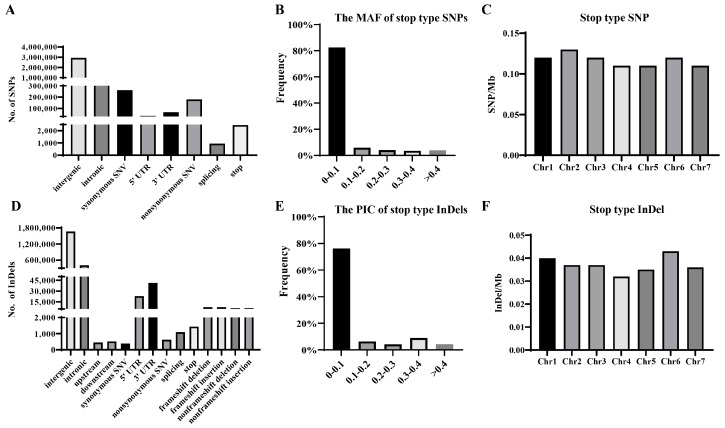
Whole-genome variations in 182 cucumber germplasms. (**A**) The number of different types of SNPs in 182 cucumber germplasms. (**B**) The MAF of stop-type SNPs. (**C**) The density of stop-type SNPs across the seven chromosomes. (**D**) The number of different InDels in 182 cucumber germplasms. (**E**) The PIC of stop-type InDels. (**F**) The density of stop-type InDels across the seven chromosomes.

The resequencing data from 182 cucumber germplasm accessions revealed 4,282,555 SNPs and 2,177,526 InDels. SNPs were annotated and classified into eight types, including intergenic, intronic, non-synonymous single-nucleotide variant, splicing, and stop. Intergenic SNPs were the most abundant, while splicing SNPs were the least common (Figure 2A). Stop-type SNPs are important for gene function, and 2462 were identified. Among them, 2079 introduced a stop codon into the gene, while 383 resulted in the loss of a stop codon (Figure 2A). Of these stop-type SNPs, 82.54% exhibited a MAF of 0–0.1, and only 17.46% showed an MAF > 0.1 (Figure 2B). The stop-type SNPs were fairly evenly distributed across all chromosomes (Figure 2C). With regard to non-synonymous SNPs, which are also important to gene function, a total of 86.94% had an MAF of 0–0.1, with only 13.06% exhibiting an MAF > 0.1 (Figure S1A). The non-synonymous SNPs were fairly evenly distributed across all chromosomes (Figure S1B).

InDels were annotated as intergenic, frameshift, and non-frameshift types, among others. Intergenic InDels were the most abundant in the cucumber genome, while synonymous SNVs were the least common. Stop-type InDels are particularly important for gene function, and 1439 of these were identified in cucumber. Among the stop-type InDels, 1332 introduced a stop codon, while 107 resulted in the loss of a stop codon (Figure 2D). Overall, 76.25% of the stop-type InDels had a PIC of 0–0.1, with only 23.75% exhibiting a PIC > 0.1 (Figure 2E). The stop-type InDels were fairly evenly distributed across all chromosomes (Figure 2F). In addition to stop-type InDels, frameshift insertions/deletions alter the protein, affecting gene function. This study identified 7453 frameshift insertions and 7459 frameshift deletions (Figure 2D). A total of 79.07% of the frameshift-type InDels had a PIC of 0–0.1, with only 20.93% exhibiting a PIC > 0.1 (Figure S1C). Frameshift-type InDels were fairly evenly distributed across all chromosomes (Figure S1D).

### 3.3. CsRR Gene Variations Are Genetically Diverse

**Figure 3:**
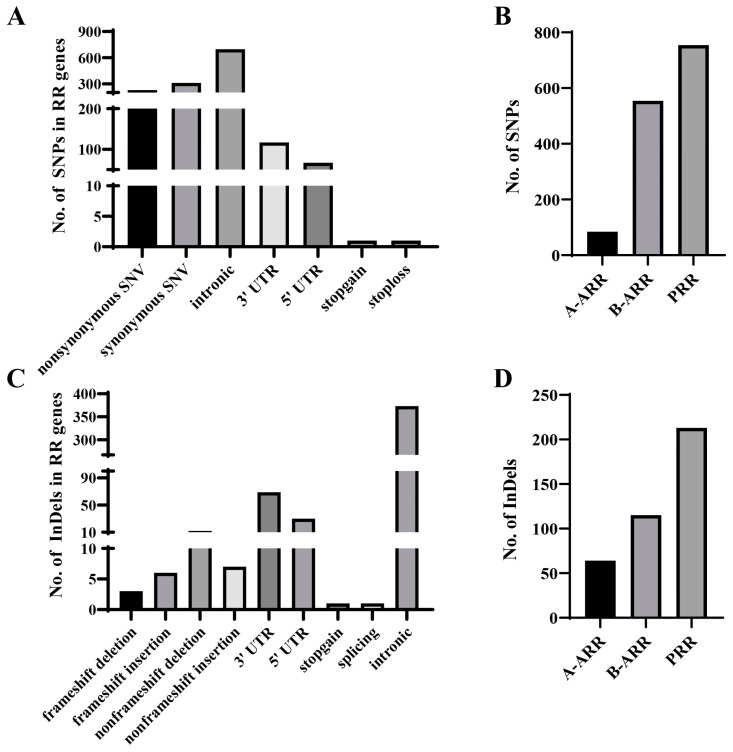
*CsRR* gene variations. (**A**) Detailed annotations of SNP variations in the *CsRR* genes. (**B**) Numbers of SNPs in the three types of *CsRR* genes. (**C**) Detailed annotations of InDel variations in the *CsRR* genes. (**D**) Numbers of InDels in the three types of *CsRR* genes.

The *CsRR* genes contained 1393 SNPs and 392 InDels, with the most variations occurring in the *CsPRRs* and the fewest in the A-ARRs (Figure 3A,C). The MAF, PIC, GD, and Ho (observed heterozygosity) values of most SNPs and InDels located in *CsRRs* were relatively low, with PIC, MAF, GD, and Ho all < 0.1 (Figure S2). Annotation of these variations revealed nine nonsynonymous SNPs in *A-CsARRs*, 114 in *B-CsARRs*, and 105 in *CsPRR* genes. Furthermore, four frameshift InDels were found in *B-CsARR* genes and five in *CsPRR* genes (Figure S3). Notably, *CsARR3* contained an SNP annotated as a ‘stop-gain’ variant, and *CsPRR5* contained a ‘stop-loss’ SNP (Figure S3A), with both genes involved in the cytokinin signaling pathway [49,50]. Additionally, a stop-gain type InDel was found in *CsPRR2* (Figure S3B), a gene reported to regulate the color of immature cucumber fruit skin [51]. Non-synonymous SNPs, frameshift mutations, and stop-gain variations significantly impact gene function. The analyses of SNPs and InDels in *CsRRs* from different populations revealed that most non-synonymous SNP and stop-type variants had low MAFs in the East Asian population, while high polymorphism was observed in the XSBN population. This suggested that the traits controlled by these genes may exhibit rich polymorphism in the XSBN population (Figure S6).

### 3.4. CsRR Gene Expression Patterns

**Figure 4:**
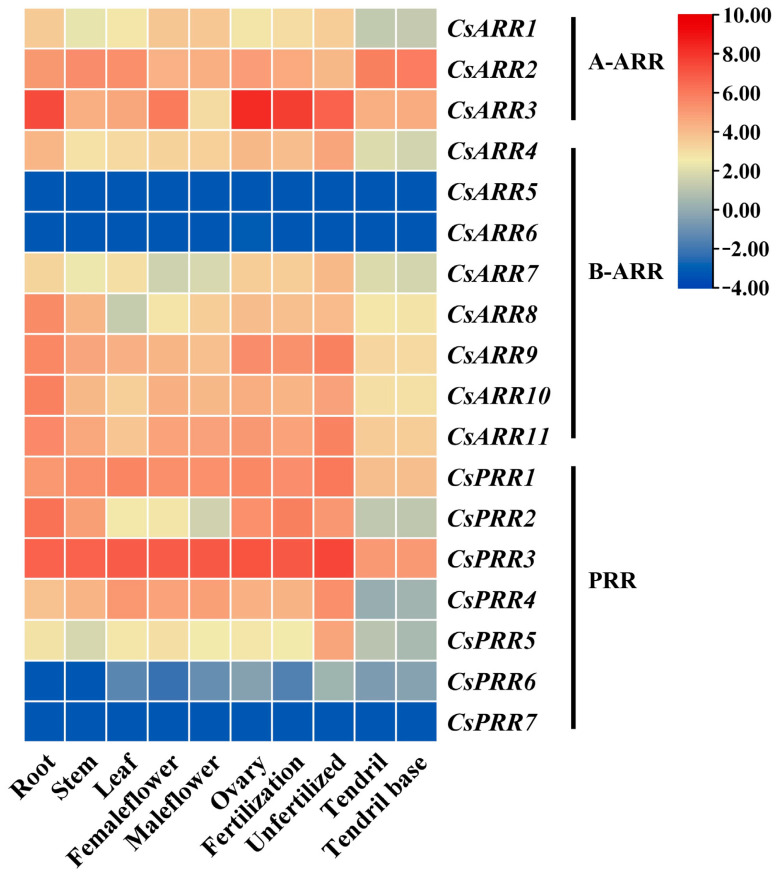
Expression profiles of 18 *CsRR* genes in various cucumber organs.

RNA-seq data from the CuGenDBv2 were used to elucidate the levels of *CsRR* gene transcription among various cucumber organs (Figure 4). *CsPRR3* exhibited high expression levels in most organs except tendrils and the tendril base. *CsARR3* was highly expressed in root, ovary, and pollinated ovary, suggesting a potential role for this gene in regulating the growth and development of these organs. *CsARR5*, *CsARR6*, and *CsPRR7* were essentially not expressed in all ten organs analyzed. *CsPRR6* was expressed at negligible levels in root and stem and exhibited low expression levels in other organs. Other *CsRR* genes were expressed in these organs but at relatively low levels. The differential expression of *CsRR* genes in various cucumber organs suggested their functions differed during the plant’s growth and development.

**Figure 5:**
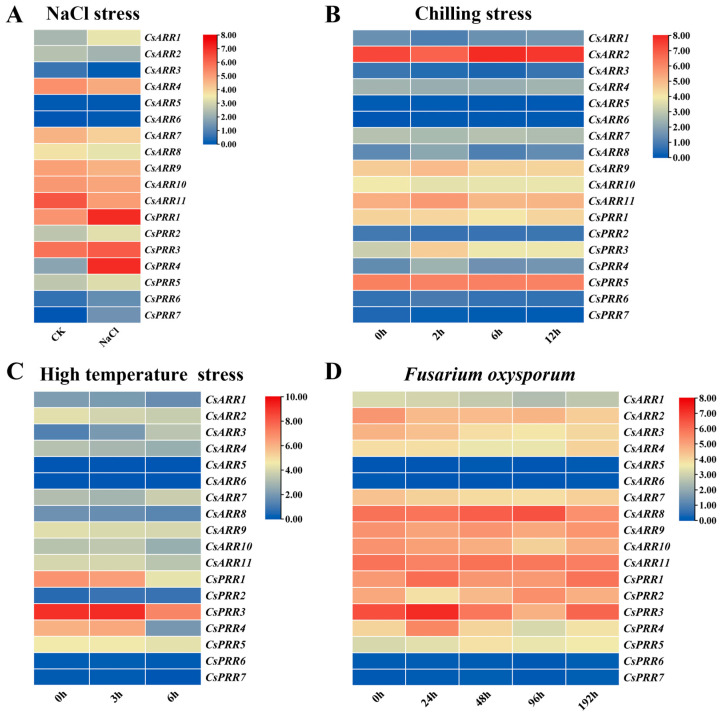
Transcriptome patterns of 18 *CsRR* genes under abiotic and biotic stresses. (**A**) NaCl stress, (**B**) chilling stress, (**C**) high-temperature stress, and (**D**) exposure to Foc.

To investigate the response of *CsRR* genes to biotic and abiotic stresses, transcriptome data from the CuGenDBv2 were further analyzed. Under NaCl treatment, the expression of *CsPRR1*, *CsPRR3*, and *CsPRR4* was significantly upregulated, while other *CsRR* genes showed varying degrees of up- and downregulation (Figure 5A). Under chilling stress, the expression of *CsARR2* and *CsPRR5* peaked at six hours, followed by a significant decrease over time under low-temperature treatment. The expression of another four genes—*CsARR8*, *CsARR9*, *CsARR11*, and *CsPRR3*—was highest two hours after the start of the chilling stress treatment, after which it decreased significantly. No significant change was observed in the expression of other *CsRR* genes (Figure 5B). Under high-temperature stress, *CsARR3* expression increased significantly with treatment duration, while that of *CsPRR1*, *CsPRR3*, and *CsPRR4* decreased significantly. The expression of other *CsRR* genes decreased but without a significant difference (Figure 5C). Finally, significant differential expression of *CsARR8*, *CsPRR1*, *CsPRR2*, and *CsPRR3* was observed following the inoculation of cucumber with Foc (Figure 5D). In summary, *CsRR* gene expression was shown to be regulated by salt stress, low- and high-temperature treatments, and Foc infection.

### 3.5. CsRR Gene Expression in Response to Foc

**Figure 6:**
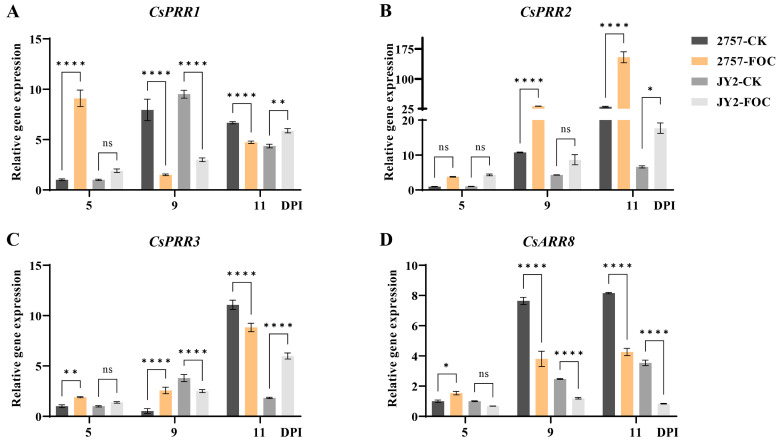
Quantitative real-time PCR analyses of *CsRR* gene expression in cucumber following inoculation with Foc. (**A**) *CsPRR1*, (**B**) *CsPRR2*, (**C**) *CsPRR3* and (**D**) *CsARR8*. The X-axis indicates the tested tissue samples. Error bars represent ± standard deviation (SD) with three biological replicates. Different asterisks above the bars indicate significant differences. (* *p* < 0.05, ** *p* < 0.01, **** *p* < 0.0001), “ns” stands for not significant.

Transcriptome data from the CsRR gene family revealed the differential expression of *CsARR8*, *CsPRR1*, *CsPRR2*, and *CsPRR3* following the inoculation of cucumber with Foc, suggesting their potential role in resistance to wilt disease. The resistant cucumber line WI2757 and susceptible line JY-2 were inoculated with Foc to examine the expression patterns of these four genes. At 11 days post-inoculation (dpi), JY-2 showed disease symptoms. RT-qPCR analyses of the four genes at 5, 9, and 11 dpi indicated the upregulation of *CsPRR1* in both the resistant and susceptible lines relative to the control at five dpi. However, *CsPRR1* expression in resistant plants was downregulated compared to the control at nine dpi, and there was no significant difference in expression between the resistant line and control at 11 dpi (Figure 6A). The expression of *CsPRR2* was upregulated in both the resistant and susceptible plant material at 5, 9, and 11 dpi compared to the control (Figure 6B). Previous studies have shown that the *PRR2* gene regulates fruit skin color [17,18], indicating that it may have a dual function in cucumber, contributing to both disease resistance and fruit skin color regulation. The expression of *CsPRR3* was significantly upregulated in the resistant line at nine dpi but significantly downregulated at 11 dpi (Figure 6C). Finally, the expression of *CsARR8* was significantly downregulated in both the resistant and susceptible plants at 9 and 11 dpi compared to the control. This differential regulation pattern suggested distinct temporal roles for these genes in plant-pathogen interactions.

## 4. Discussion

The RR gene family has been studied extensively in *Arabidopsis* [52], tomato [53], tobacco [54], rice [55], maize [56], and citrus [57], but the functional characterization of these genes in cucumber has not been reported. In this study, 18 *CsRR* genes were identified using bioinformatics and classified into three groups according to their protein structures: *A-ARRs* (3), *B-ARRs* (8), and *PRRs* (7).

A genetic re-sequencing analysis of 182 cucumber germplasm lines identified key mutation sites in the RR gene family, including three stop mutations, 228 non-synonymous mutations, and nine frameshift mutations. Stop mutations play a critical role in plant growth and development [42]. For example, *AtARR3* plays a cytokinin-independent role in the regulation of the circadian rhythm [58]; meanwhile, *AtPRR5* interacts with and stimulates ABI5 to modulate abscisic acid signaling during seed germination [5], the circadian clock component *OsPRR5* modulates photoperiodic flowering through transcriptional regulation of florigen genes in rice [59]. *CsPRR2* is hypothesized to regulate the color of immature fruit skins [16]. Recent molecular genetic analyses have demonstrated that the C1 locus governing fruit pigmentation in *Capsicum annuum* is functionally associated with the *PRR2* gene [60]. Additionally, the PIC, MAF, GD, and Ho values of *CsRR* genes with SNP and InDel variants, within the range 0.1–0.2, were found to exceed 75%, indicating high genetic diversity, complex genetic outcomes, and suitability for genetic research.

Tissue-specific gene expression is crucial for understanding gene function [61]. Therefore, the transcriptome data for *CsRR* genes in ten cucumber organs were analyzed. The results indicated that *CsRRs* were involved in cucumber growth and development, with distinct biological roles in various tissues. In *Zanthoxylum armatum*, the tissue expression patterns of *ZaRR* family members exhibited significant variation. For instance, *RR11* was specifically expressed in young and mature leaves; *RR12* was highly expressed in roots and mature leaves; and *RR21* showed high expression in roots, stems, mature leaves, and leaf buds [62].

Studies have demonstrated the involvement of RRs in the response to abiotic stress, as well as their roles in growth and development. Jain et al. observed the expression of *OsRR6* in rice seedlings to be significantly upregulated under salt and low-temperature stress [40]. In the present study, *CsRR* gene expression in cucumber was modulated under abiotic stress conditions, suggesting that these genes may be critical to stress resistance. Furthermore, the functional involvement of RR gene family members in plant responses to biotic stresses was analyzed. Following Foc inoculation, differential expression patterns were observed between resistant and susceptible cucumber cultivars. The differential expression of *CsPRR2*, *CsPRR1*, *CsPRR3*, and *CsARR8* following Foc inoculation suggested potential roles for these genes in the response of cucumber to wilt disease. *CsPRR1* expression was significantly upregulated in both resistant and susceptible lines compared to the CK at five dpi. However, in resistant lines, transcript levels declined below CK values by nine dpi and returned to CK-equivalent levels by 11 dpi. *CsPRR3* exhibited genotype-dependent induction kinetics, showing robust upregulation in resistant lines at nine dpi and delayed activation in susceptible lines, with peak expression only observed at 11 dpi. 

*CsARR8* was persistently suppressed in both genotypes, exhibiting significant downregulation at 9 and 11 dpi relative to the CK. Notably, *CsPRR2* was upregulated in resistant and susceptible lines at 5, 9, and 11 dpi, indicating its involvement in early and sustained defense. In rice, the *OsPRR1* gene may play a regulatory role in photoperiodic flowering, potentially integrating circadian clock signals with light-dependent pathways to control floral transition [41]. Jeong et al. found that the fruit color locus C1 in *Capsicum* is associated with *PRR2* [42]. In *A. thaliana*, *PRR3* expression and function are more prominent in the vasculature [43], suggesting a mechanism that fine-tunes the plant’s clock in this tissue. *ARR8* is involved in cytokinin signal transduction and functions as a negative regulator [44]. Based on our experimental results, *CsARR8*, *CsPRR1*, *CsPRR2,* and *CsPRR3* have been demonstrated to collectively play a critical regulatory role in the growth and development of cucumber. Based on the analysis of transcriptome data, significant differences in the expression levels of four *CsRR* genes were found following inoculation with Foc. Functional characterization revealed that *CsPRR2* carries an important stop-gain mutation and exerts dual regulatory roles. In addition to regulating the color of immature fruit skin, it also mediates basal defense against Foc. This systematic exploration of the molecular mechanisms by which *CsRR* genes regulate cucumber growth and development, as well as its responses to biotic and abiotic stresses, provided an important theoretical foundation for the genetic improvement of disease and stress resistance traits in cucumber.

## Data Availability

Data are contained within the article and Supplementary Material.

## Supplementary Materials

The following supporting information can be downloaded at: https://www.mdpi.com/article/10.3390/genes16040409/s1, Figure S1: The genetic diversity and mutation density of nonsynonymous- type SNPs and frameshift-type InDels in 182 cucumber germplasm; Figure S2: The genetic diversity of SNPs and InDels in the 18 *CsRR* genes; Figure S3: The important mutation located in three types of 18 *CsRR* genes; Figure S4: The genetic analysis of nonsynonymous-type SNPs; Figure S5: The genetic analysis of frameshift-type InDels; Figure S6: The MAF of nonsynonymous type SNPs in the 18 *CsRR* genes. Table S1: Protein sequences of the CsRR genes family; Table S2: A list of genome-wide mutations and the number of mutations in the CsRR gene family; Table S3: List of transcriptome data; Table S4: qRT-PCR primer sequences.
