# Supplementary material for: Resequencing and Transcriptome Analyses Reveal Variations and Expression Patterns of the RR Gene Family in Cucumber
Source: Genes (Basel). 2025 Mar 31;16(4):409. doi: 10.3390/genes16040409 (PMC12027353; doi:10.3390/genes16040409)
Supplement: Supplementary file 1 [file genes-16-00409-s001.zip › Supplementary Figures.pdf]

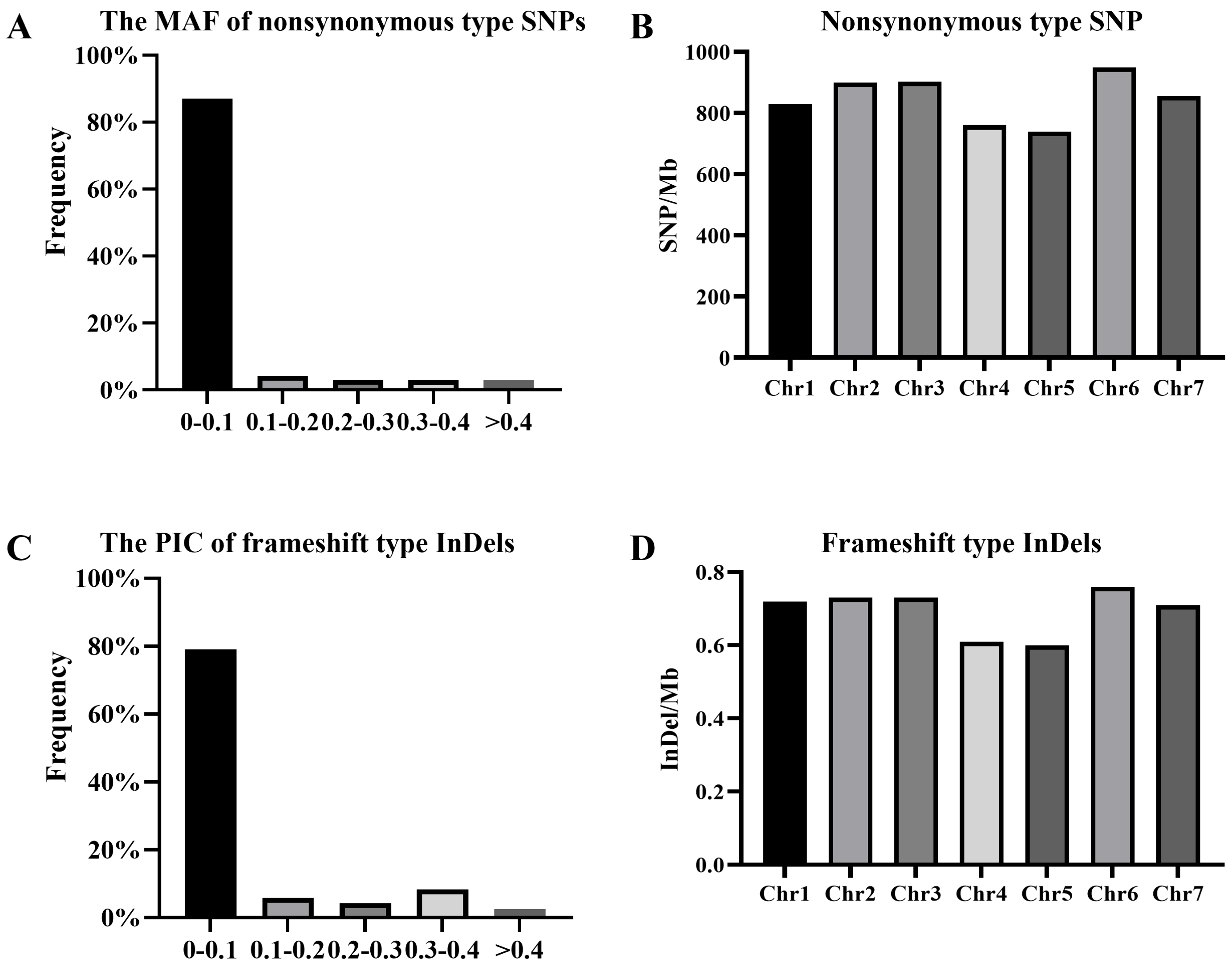

Figure S1. The genetic diversity and mutation density of nonsynonymous- type SNPs and frameshift-type InDels in 182 cucumber germplasm. (A) The MAF of nonsynonymous-type SNPs. (B) The SNP density across different chromosomes. (C) The PIC of frameshift-type InDels. (D) The InDel density across different chromosomes.

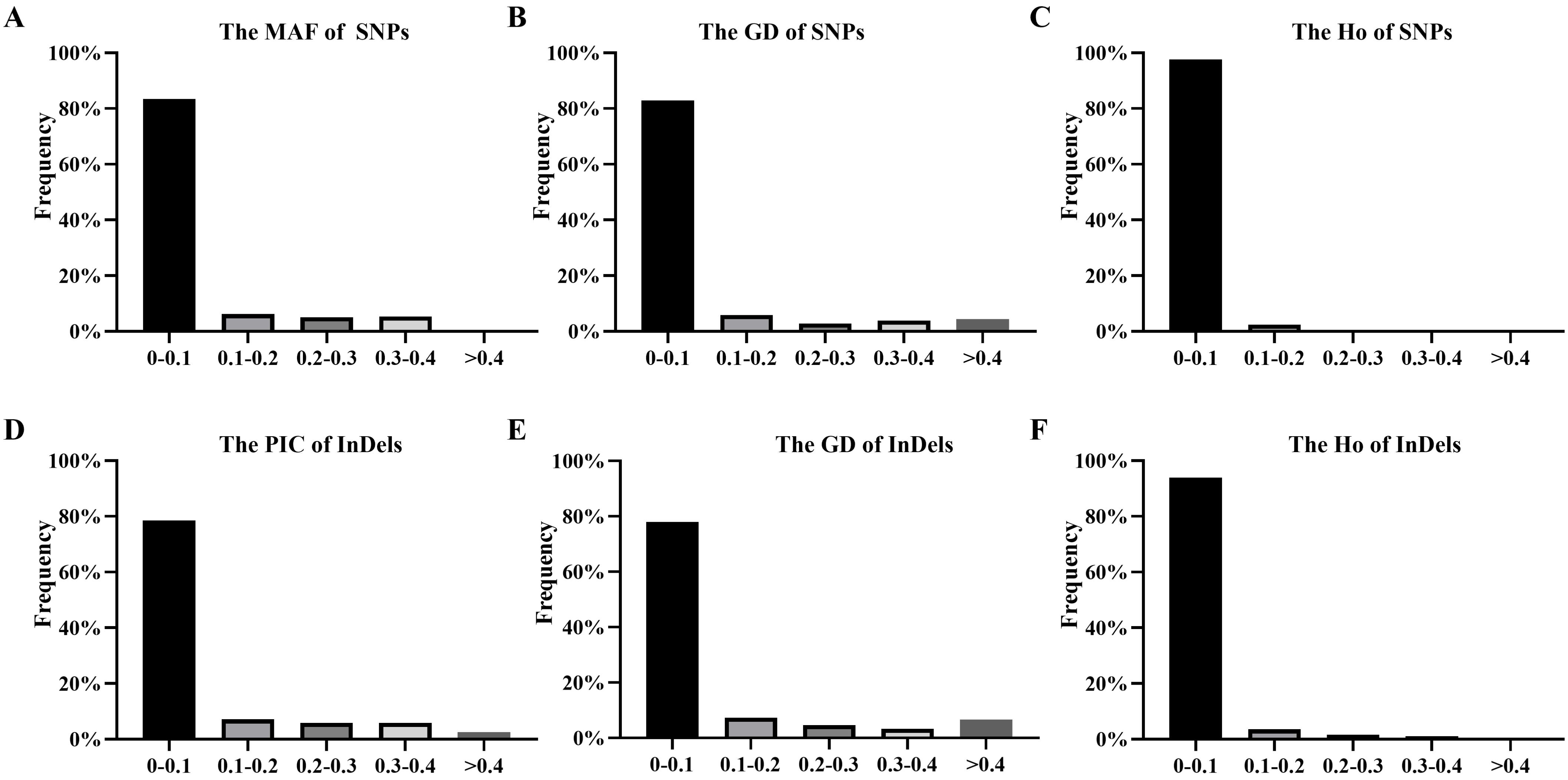

Figure S2. The genetic diversity of SNPs and InDels in the 18 *CsRR* genes. The (A) MAF, (B) GD, and (C) Ho of SNPs located in 18 *CsRR* gene. The (D) PIC, (E) GD, and (F) Ho of InDels located in the 18 *CsRR* genes.

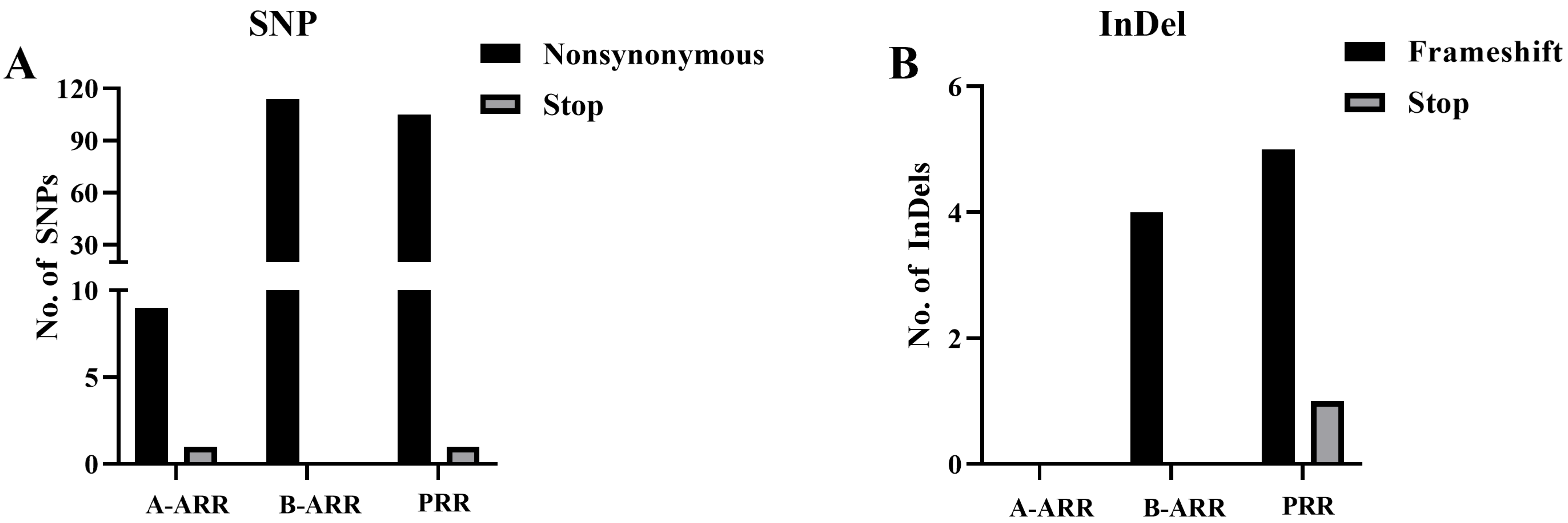

Figure S3. The important mutation located in three type of 18 *CsRR* genes. (A) The number of nonsynonymous and stop-type SNPs in A-ARR, B-ARR, and PRR. (B) The number of frameshift and stop-type InDels in A-ARR, B-ARR, and PRR.

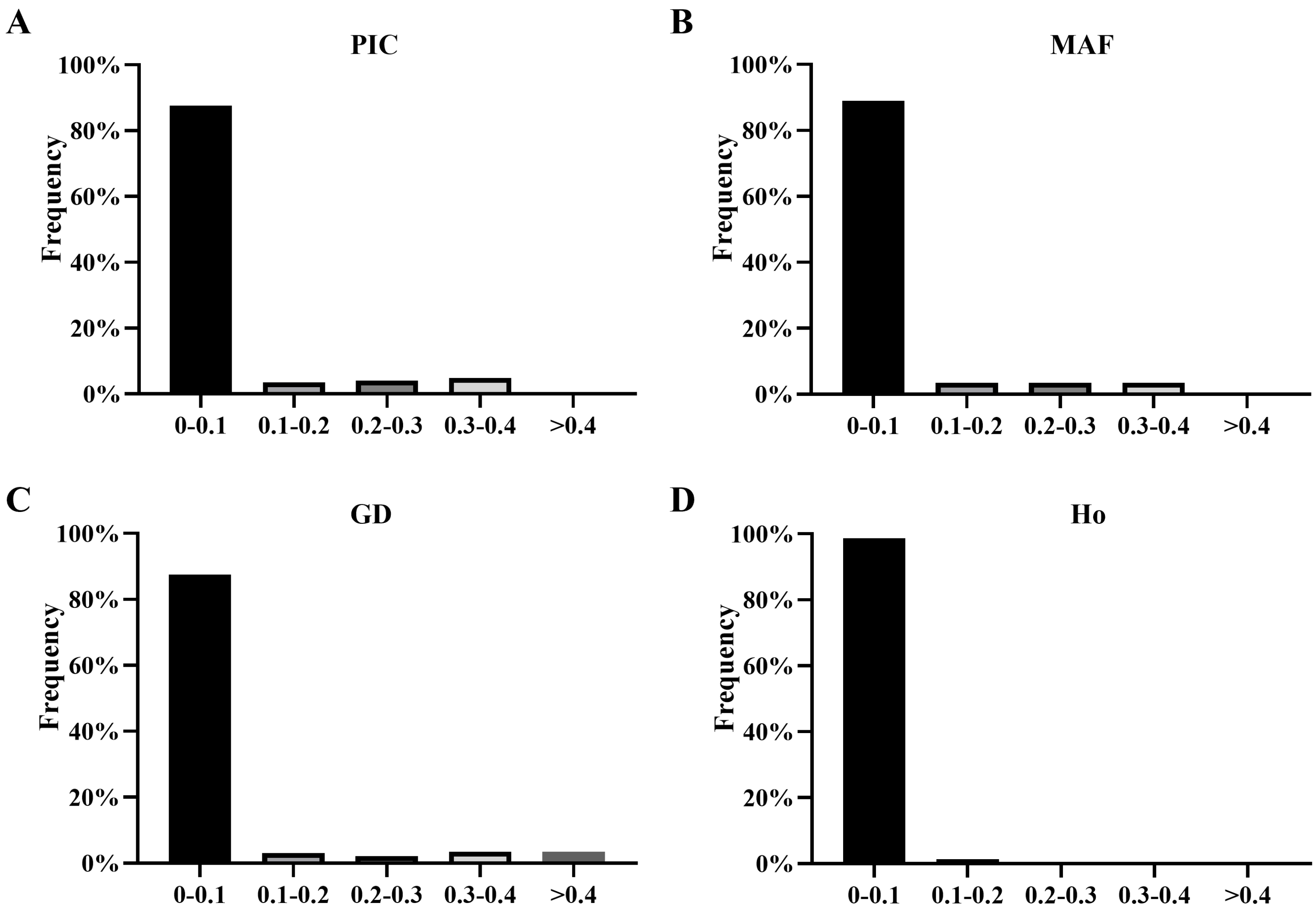

Figure S4. The genetics analysis of nonsynonymous-type SNPs, including (A) PIC, (B) MAF, (C) GD, and (D) Ho.

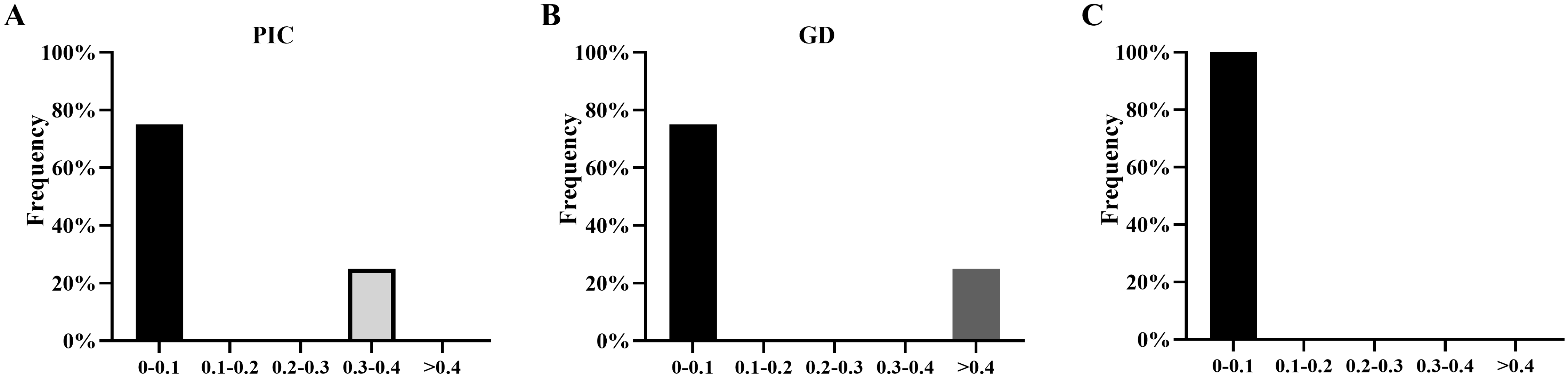

Figure S5. The genetics analysis of frameshift-type InDels, including (A) PIC, (B) GD, and (C) Ho.

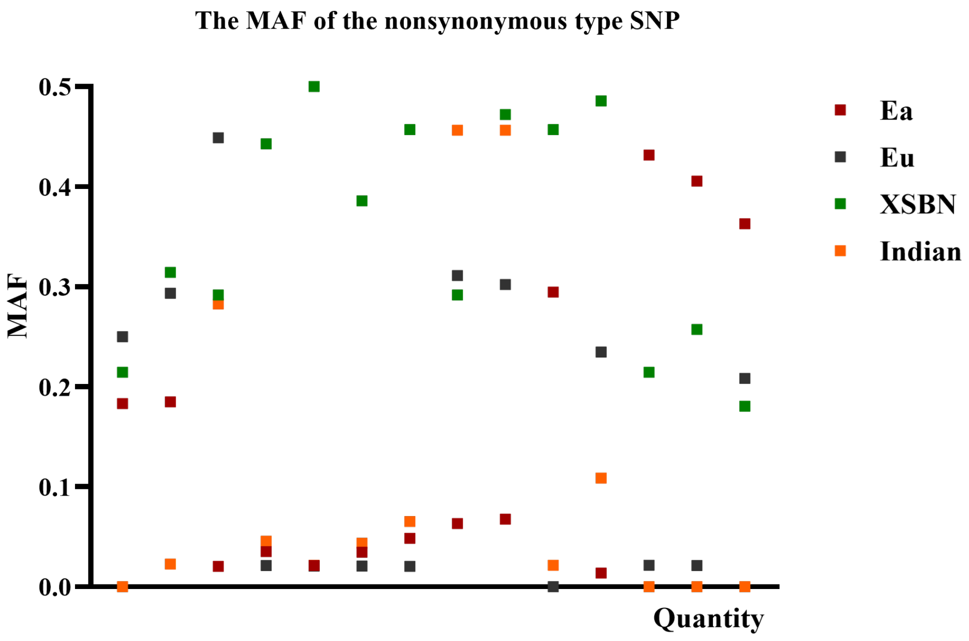

Figure S6. The MAF of nonsynonymous type SNPs in the 18 *CsRR* genes. The color red, blue, green, and orange represent East Asian (Ea), European (Eu), XiShuangBanNa(XSBN), and Indian populations, respectively.
